# Impact of gold mining on the water quality of the lom river, Gankombol, Cameroon

**DOI:** 10.1016/j.heliyon.2022.e12452

**Published:** 2022-12-21

**Authors:** Mouhamed Ngounouno Ayiwouo, Fadimatou Ngounouno Yamgouot, Luc Leroy Ngueyep Mambou, Sifeu Takougang Kingni, Ismaila Ngounouno

**Affiliations:** a Department of Mining Engineering, School of Geology and Mining Engineering, University of Ngaoundere, P.O. Box 115, Meiganga, Cameroon; b Department of Earth Sciences, Faculty of Sciences, University of Ngaoundere, P.O. Box 454, Ngaoundere, Cameroon; c Laboratory of Material Sciences, Department of Physics, Faculty of Sciences, University of Yaoundé 1, P.O. Box 812, Yaoundé, Cameroon; d Department of Mechanical, Petroleum and Gas Engineering, National Advanced School of Mines and Petroleum Industries, University of Maroua, P.O. Box 46, Maroua, Cameroon

**Keywords:** Gold mining, Lom river, Hydrochemical assessment, Multivariate analysis, Metal pollution, Geostatistical modeling

## Abstract

The aim of this paper is to assess the impact of gold mining on the water quality of the Lom River at Gankombol (Adamawa Cameroon). Forty-eight (48) water samples are systematically collected during the dry and the rainy season. These water samples are characterized to determine the physico-chemical parameters, major ions and metals. A local geological study is conducted to determine the relationship between the geological units encountered and water contamination. Hydrochemical assessment, multivariate statistical analysis (MSA) and geostatistical modeling (GM) are used to assess contamination. The results indicate that the waters of the Lom River draining the gold mining site are acidic to neutral (5.3–6.9), very turbid (117–510 NTU) with high concentrations of suspended solids (22.89–471 mg/L). The mean concentrations of Fe, Pb, and As exceed the limits set by the World Health Organization (WHO) standards. Pb, As, Cd and Hg concentrations decrease in the rainy season mainly due to dilution by rainwater. The predominant water type is Ca–Mg–HCO_3_. This surface water is unsuitable for drinking purpose (997.5, Water Quality Index >300) with high level of metal pollution. MSA reveal strong linear correlations between EC-TDS, EC-Na^+^, TDS-Na^+^, Pb–As, Cl^−^ –SO_4_^2-^ and TSS-Cd suggesting that the correlated parameters can have common origin. Finally, GM reveal that the lowest values of metals and pollution indices are found upstream of the gold mining site. The weathering of geological units encountered, mining activities and seasons have a major influence on the water quality. Therefore, it appears that decision-makers must take immediate action to decrease pollution and adopt suitable and sustainable remedial solutions.

## 1 Introduction

The development of methods for improving water quality, distributing drinking water to people, and conserving ecological systems are significant problems in answering questions about sustainable development. River and environmental pollution has long been a source of concern, and it continues to grow in relevance in today's globe. Rivers have been researched extensively in comparison to other bodies of water in terms of water quality, which is not surprising considering that humans and aquatic species interact with them frequently and are thus more susceptible to contaminants transported there (Falconer et al., 2012). Water is the most important, plentiful, and beneficial natural resource on the planet, and life cannot exist without it. It is a requirement of human life and, as such, should be of the highest possible quality. Water's quality is determined by its physico-chemical and biological qualities (Diersing, 2009). The quality of water is more significant than the quantity of this resource in the planning and management of water delivery to communities. Pure water is required for good quality consumption (Namita and Alka, 2017). The state of water, as defined by its quality, is critical to biotic species' demands and/or any human need or purpose (Johnson et al., 1997).

Several investigations on the impact of anthropogenic activities on water quality of surface water have been conducted around the world (Kaizer and Osakwe, 2010; Malik and Nadeem, 2011; Thakur et al., 2015; Asare-Donkor et al., 2018; Dippong et al., 2018; Umer et al., 2020; Dippong et al., 2020; Roș;ca et al., 2020; Maliqi et al., 2020; Gautam et al., 2021; Karmakar et al., 2021; Matta et al., 2022). Other studies have shown the specific impact of mining on water resources (Attua et al., 2014; Aikins et al., 2015; Cidu et al., 2013; Mcdonald et al., 2015; Goix et al., 2019; Corredor et al., 2021). Similar studies have been carried out in coal mines in India (Palit et al., 2017), Korea (Nigam et al., 2015) and China (Liu et al., 2020) and in Africa, particularly in Nigeria (Isa et al., 2019; Ekwule et al., 2019; Benibo and Sha’ato, 2020), in Ghana (Acheampong et al., 2013; Banunle et al., 2018). In Bolivia in the Val Milluni, the impact of heavy metals on surface waters and sediments from mining waste using geochemical, mineralogical and hydrochemical approaches was investigated (Aranguren, 2008). Finally, studies have been carried out specifically in abandoned mines, particularly in Morocco (Hakkou et al., 2005; Baghdad et al., 2012; Chakraaoui, 2015), in abandoned iron ore extraction sites in Kuala Lipis and Bukit Ibam (Madzin et al., 2017) and in abandoned mines in Selangor (Hamzah et al., 2020). All these studies have shown that small, medium or large scale mining operations have (during or after) environmental consequences on water resources. These impacts that become a problem local communities living in this environment.

In Cameroon, previous research have demonstrated that artisanal and semi-mechanized mining activities had an impacts on water resources (Rakotondrabe et al., 2017a, b; Mambou et al., 2020a, b). These impacts were manifested by physical pollution and the presence of metals such as Pb, Cr, As, Cd and Hg and ligand (CN) in surrounding waterways. The concentrations of these metals in waters were higher than the standards of the World Health Organization (WHO). Few studies have been done in the Adamawa region to investigate the impacts of mining activities on surface water (Ayiwouo et al., 2020, 2021). In comparison to the studies cited above, the novelty of this study lies in the assessment of the spatial and temporal impact of gold mining activities at Gankombol with a greater sample size; the integration of a local geological study; more methods of water quality assessment and the use of multivariate statistics and geostatistical modeling. This in order to provide the most updated representation of contamination or pollution of Lom River draining the gold mining site. In addition, the present study hopes to demonstrate that mining activities (artisanal and semi-mechanized) have negative environmental impacts on the Lom River at Gankombol. This is in order to help decision makers to take immediate measures to reduce the negative impacts of these activities for providing solutions for a rational and sustainable exploitation of gold mining sites.

The objectives of this study were to: (i) conduct a spatio-temporal study of the impact of gold mining on the quality of the Lom River; (ii) determine the correlation between the geological units encountered in the study area and water contamination; (iii) assess the global quality and metal contamination status of this surface water (iv) determine the potential source of contamination using multivariate statistics and (v) predict the contamination using geostatistical approach. The obtained results will be useful to provide a decision support document for the authorities with a view to reducing the risk of water pollution near gold mining sites and increasing their capacity to manage potentially polluted sites.

## 2 Materials and methods

### 2.1 Study area

**Figure 1:**
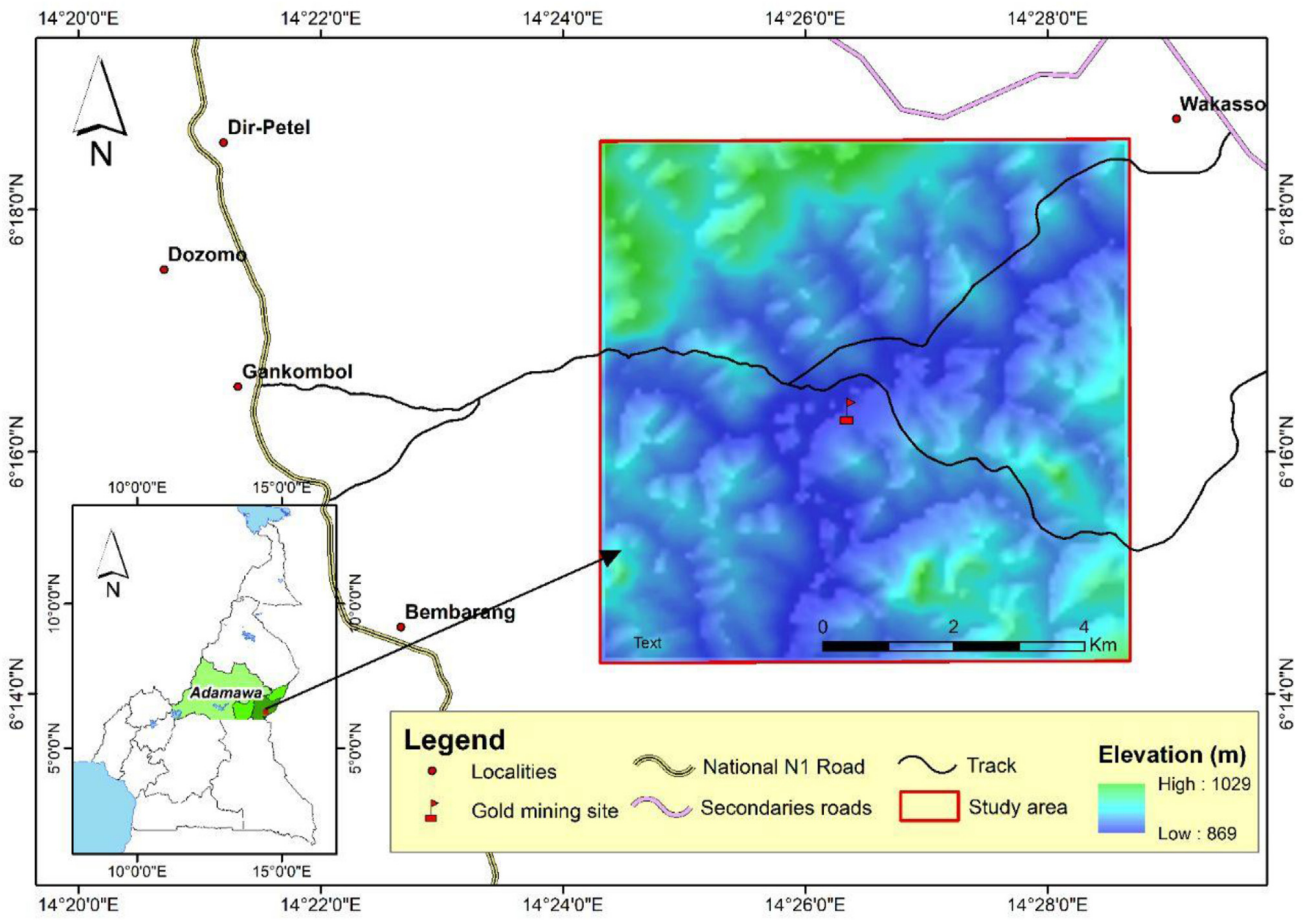
Location map of the study area and the gold mining site.

The study area is centered on the village of Gankombol in the district of Meiganga, department of Mbéré and Adamawa region (Figure 1). It is located about 20 km from the town of Meiganga. It is accessible by paved road on the N°1 national road from Meiganga, followed by an unpaved secondary axis of about 6 km from the village of Gankombol. The area of interest is located northeast of the village of Gankombol at the coordinate points: A (6.308992°; 14.405018°) B (6.309964°; 14.477550°) C (6.237489°; 14.405379°) and D (6.237259°; 14.477774°). The gold mining site is located at geographical coordinates N 6.272740° and E 14.434460°. It is drained by the Lom River. Roughly rectangular with a length of 8.5 km and a width of 7.56 km, the area of interest covers just over 64.30 km^2^, with an altitude varying from 875 m to 1025 m.

The climate of the study area is monomodal Sudanese tropical with two seasons: a dry season from November to April and a humid season the rest of the year. Throughout the year, Gankombol has a mean temperature of 23.1 °C. The mean annual rainfall is 1551 mm. December is the driest month, with only 4 mm of rain. The wettest month is September, with a mean rainfall of 256 mm. With a mean temperature of 24.8 °C, March is the hottest month of the year. With a mean temperature of 21.9 °C, July is the coldest month of the year (Climate-Data.org, 2021).

The relief corresponds to a plateau of altitude between 871 and 1025 m which are relatively very high. The highest areas are located in the North - West with altitudes between 933.91 and 1012.36 m while the lowest areas are located in the center with altitudes between 872.69 and 894.53 m, in particular on the Lom River and the mining area. The Lom River is the principal watercourse in the study area and runs through the mining area, with the most of the mining taking place on the East side of the river. The Lom River is frequently diverted and used to wash ore during mining.

The gold mining site of Gankombol was chosen for a variety of reasons. These include, among others: The degree of gold mining observed on the site; very little physico-chemical and metallic characterization work on the waters of gold mining sites in the region; the absence of physico-chemical and metallic characterization works on the waters of the Lom River draining the gold mining site gold at Gankombol; the lack of information on the local geological context and the easy access to the site by fairly maintained village tracks.

### 2.2 Geology of the study area

**Figure 2:**
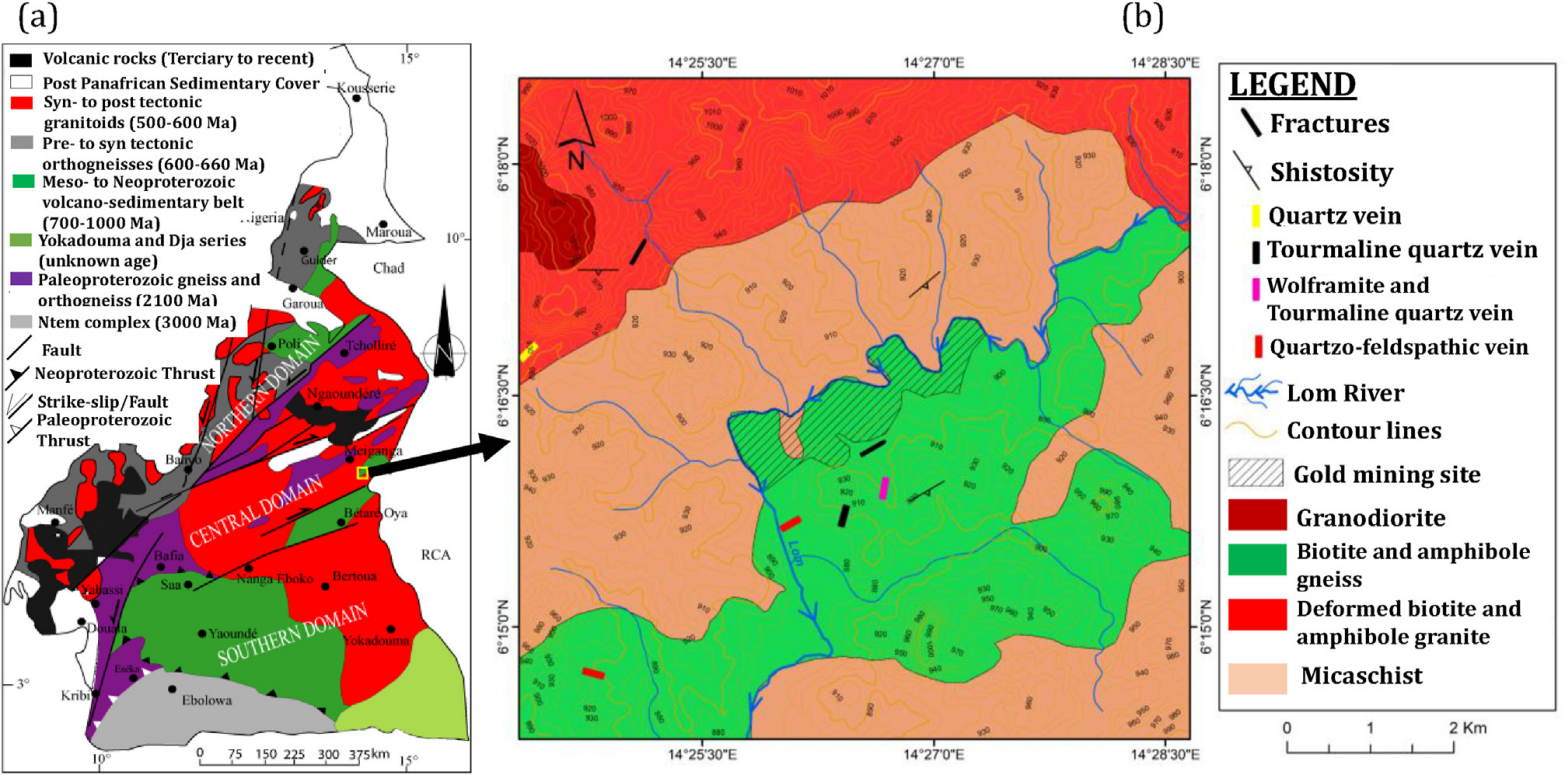
(a) Geological map of Cameroon showing the three domains of the Central African Belt and the major bordering lithotectonic structures (modified from Nkoumbou et al., 2014). TBSZ, Tcholliré– Banyo Shear Zone; CCSZ, Central Cameroon Shear Zone; SSZ, Sanaga Shear Zone and (b) Geological map of the study area.

The study area is located along the Lom Group, and belongs to the Adamawa-Yadé domain or central Cameroon domain of the Central African Orogenic belt (CAOB) in Cameroon (Figure 2a). This domain extends from the north of the Bafia group to the south of the Tcholliré-Banyo shear zone [(TBSZ); Toteu et al., 2004; Nkoumbou et al., 2014; Tchakounté et al., 2017)] also called the Kekem-Fotougni shear zone (Kwékam et al., 2020) and it is made of: (1) Migmatitic gneisses and metasedimentary rocks (Penaye et al., 2004); (2) Metavolcano-sedimentary rocks belonging to the Lom series, recrystallized under low-to medium-grade metamorphic conditions (Soba et al., 1991; Ngako et al., 2003; Toteu et al., 2006); and the amphibolite from Mbé—Sassa-Berci (Saha-Fouotsa et al., 2019); (3) Syn-to late-orogenic weakly deformed granites showing calc-alkaline to shoshonitic affinity (Toteu et al., 2001; Tchameni et al., 2006; Saha-Fouotsa et al., 2019). The Lom group consists of metatuffs, volcaniclastic and sedimentary-derived schists, staurolite–garnet micaschists, and quartzites with local conglomerate layers (Soba et al., 1991). The Lom group is known for its numerous gold occurrences (Assah, 2010; Ndonfack et al., 2021). Gold in this area are claissified as orogenic gold and the main mineral paragenesis consist of pyrite, chalcopyrite, sphalerite, galena and arsenopyrite (Suh et al. 2006; Vishiti et al., 2019; Ndonfack et al., 2021). The geological map of the study area is presented in Figure 2b.

A local geological study was carried out in order to identify the lithological units in the study area. The main lithological units identified in the field are reported on the geological sketch map of Figure 2b. The metamorphic units consist of biotite-amphibole gneisses and micaschists. The magmatic units comprise mainly plutonic rocks made up of granodiorites and deformed biotite-amphibole granites. Metamorphic and plutonic units are cross-cut by many veins with variable length, width and compositions: quartz and quartzo-feldspathic veins, tourmaline (Fe-rich) and tourmaline (Fe-rich)-wolframite bearing quartz veins. Rocks of Gankombol are affected by intense hydrothermal alteration made of silicification, sulfuration, feldsptahisation, chloritisation, hemantization and carbonatation. The intensity of alteration increases in the vicinity of tourmaline and tourmaline-wolframite quartz veins.

### 2.3 Sampling and analytical procedures

**Figure 3:**
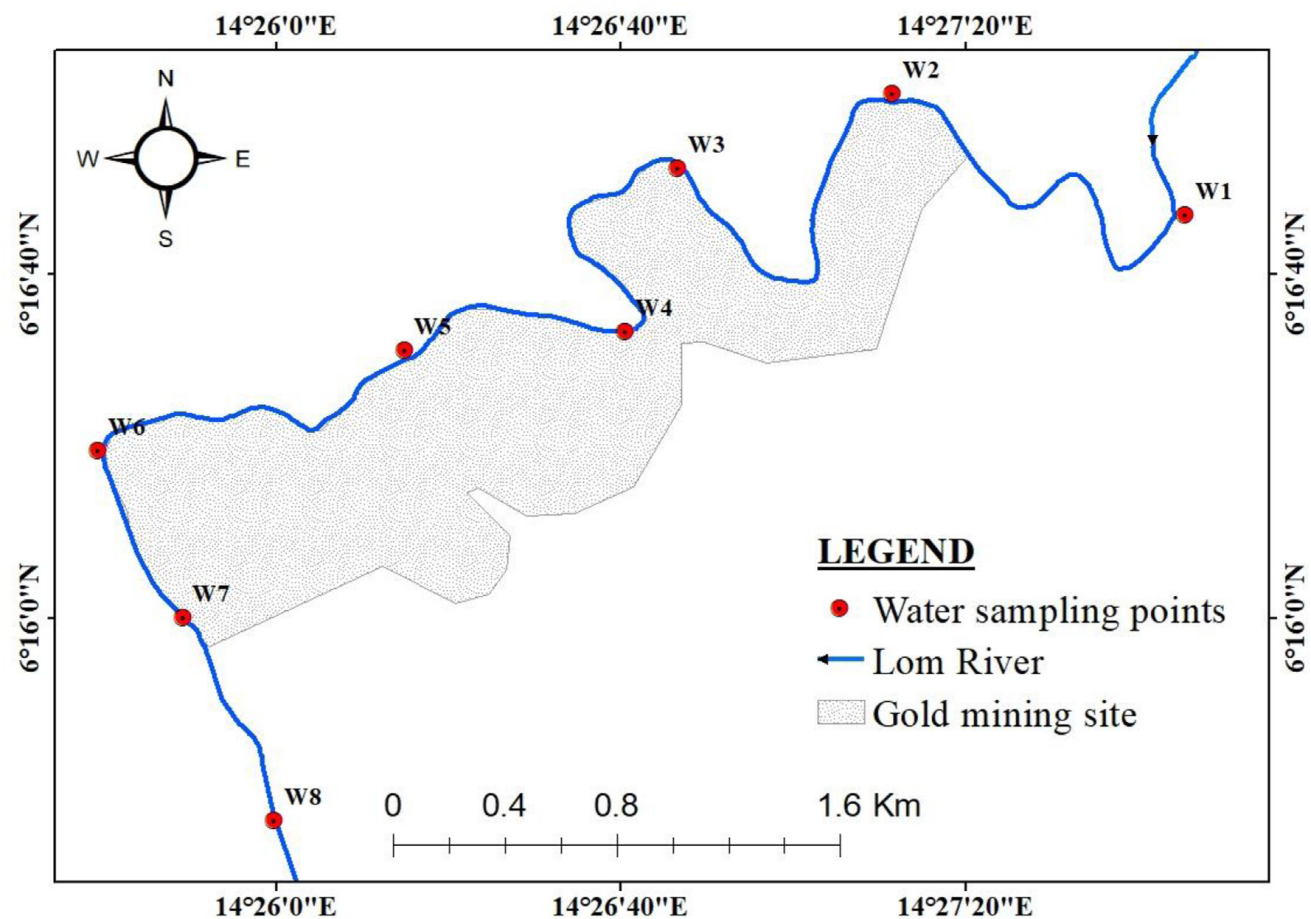
Sampling map.

The water sampling points were chosen based on their accessibility, representativeness, and closeness to gold mining operations. Six (06) sampling campaigns were conducted during the dry season (February, March and April 2021) and the rainy season (May, June and July 2021). A total of forty-eight (48) water samples were collected. Eight (08) samples were taken once a month for six (06) months along the studied section of the Lom River. Samples W1 and W8 were taken upstream and downstream of the mining site in order to assess the impact of gold mining activities on the water of the Lom River. Samples W2 to W7 were collected in the mining site. The sampling map is shown in Figure 3.

The sampling was performed according to the standard procedures (European Standards – SR ISO 5667-23, 2011; European Standards – SR ISO 5667-3, 2013). The water samples were collected in clean polyethylene bottles. The sampling was completed by plunging the bottle into the water source at 50 cm depth and allowing it to fill without air, after the bottle was rinsed with the water sample. Water samples intended for major ion and metal analyses were filtered in the field through 0.45 μm pore-size acetate cellulose filters and immediately acidified to pH < 2 with ultrapure nitric acid (HNO_3_). All samples were meticulously labeled and preserved in a cooler at 4 °C protected from the sunlight until they were transported to the laboratories for analyses.

The water samples were analysed for a set of physico-chemical, physical and chemical indicators to investigate the environmental changes that may affect the water quality. The main physico-chemical parameters, such as hydrogen potential (pH), electrical conductivity (EC), total dissolved solids (TDS), and temperature (T°) were measured in situ with a Hanna HI991300 – HI991301 portable equipment that had previously been calibrated. All glassware were washed with detergents, rinsed with 10% nitric acid, and then rinsed three more times with deionized distilled water. The ultrapure water with a resistivity of 18.2 MΩ was employed in the blank and the standard samples. Alkalinity (Alk), turbidity (Tu) and total suspended solids (TSS) were analysed in laboratory. These parameters were determined in the laboratory of Geochemical and Water Analysis of the Geological and Mining Research Institute of Yaounde (Cameroon). The alkalinity was calculated using Eq. (1) (Gran, 1952):

(1) $$Alk.\left(\frac{\mu mol}{L}\right)=\frac{C_{a}\times V_{e}}{V_{o}}\text{,}$$

where $C_{a}$ is the normality of the acid; $V_{o}$ the initial volume of the sample and $V_{e}$ the equivalent volume.

The Turbidity was measured by using a Hach DR/3900 spectrophotometer at a wavelength of 450 nm. The TSS was determined using the weight difference between the filter before and after filtering. The filter must be dry for the final measurement. After drying in a 105 °C oven, the filters were weighed on a balance after passing through a desiccator. The result can finally be represented in mg/L of TSS by taking into consideration the volume filtered for each filter. The TSS was determined using Eq. (2).

(2) $$TSS=\frac{M2-M1}{V}\times 100,$$

where TSS is the total suspended solids in mg/L; M1 the mass in mg of the filter before filtration; M2 the mass in mg of the filter after filtering and drying, and V the volume of filtered water in mL.

The chemical elements analysed in water samples were major ions (Na^+^, Ca^2+^, Mg^2+^, K^+^, NH_4_^+^, Cl^−^, F^−^, NO_3_^-^, PO_4_^3-^, SO_4_^2-^ and HCO_3_^-^) and metals (Fe, Pb, As, Cd and Hg). The choice was made on Pb, As, Cd and Hg because they are highly toxic and have no physiological function on the human organism. For Fe, the choice was made on this metal because it is regularly associated with gold satellite minerals such as pyrite in the country’s gold bearing context and the soils of the study area are also ferralitic (Segalen, 1967). It was therefore interesting to monitor the iron contamination of these waters. Major cations (Na^+^, Ca^2+^, Mg^2+^, K^+^ and NH_4_^+^) analysis was performed by ion-chromatography using a Thermo Scientific Dionex ICS-90 ion-chromatograph with an error of ≤5%. Major anions (Cl^−^, F^−^, NO_3_^-^, PO_4_^3-^, SO_4_^2^ and HCO_3_^-^) analysis was performed using the Thermo Scientific Dionex ICS-1100 ion-chromatograph. These analyses were carried out in the laboratory of Geochemical and Water Analysis of the Geological and Mining Research Institute of Yaounde (Cameroon). To check the accuracy and dependability of the analysis results of major ions, the charge balance calculation was performed using Diagrams software. It enables to evaluate the fractional difference between total cations and anions. The mean charge balance was 1.8% and less than 5%. The Inductively Coupled Plasma Optical Emission Spectrometry (ICP-OES) was used to analyse metals after nitric acid digestion. The Optima 8000 spectrometer was utilized in the experiment. This analysis was carried out at the laboratory of the International Institute of Tropical Agriculture (IITA) of Yaounde (Cameroon).

### 2.4 Assessment of the global quality of water

#### 2.4.1 Hydrochemical analyses

To determine the hydrochemical nature of the water and the suitability for irrigation use, hydrochemical analyses were carried out. The Piper, Wilcox, and Riverside diagrams were determined using Diagrams software.

#### 2.4.2 Water quality index (WQI)

The WQI is a mathematical method for reducing a huge amount of measurable information about water into a single piece of information (value) in an intelligible format (Štambuk-Giljanović, 1999). It was used to assess overall water quality and the viability of using it for human consumption by assessing the impact of specific water parameters (Mitra et al., 2006; Yadav et al., 2015). The calculation of WQI involves five steps as follows: (i) assigning weight (wi), (ii) relative weight ($W_{i}$), (iii) quality rating ($q_{i}$), (iv) sub-index (SI) and (v) summation of sub-index (WQI) (Anwar et al., 2015; Ewaid and Ali, 2017). To calculate the WQI of drinking water, each parameter was allocated by a certain numeric value, i.e. assigning weights ($w_{i}$), in a range of 1 (least) to 5 (highest). The ranking is based on the parameter significance to the water quality, where the maximum weight of ‘‘5’’ was designated to the most significant parameters (Table 2) (Yidana and Yidana, 2010; Varol and Davraz, 2015). Second, the relative weight ($W_{i}$) of the chemical parameter was computed by using Eq. (3):

(3) $$W_{i}=\frac{w_{i}}{\sum_{i=1}^{n}w_{i}},$$

where $W_{i}$ is the relative weight, $w_{i}$ the weight of parameter, n the number of parameters. Third, the quality rating ($q_{i}$) of each parameter was estimated using Eq. (4):

(4) $$q_{i}=\frac{C_{i}}{S_{i}}\times 100,$$

where $q_{i}$ is the quality rating, $C_{i}$ the mean concentration (mg/L) of respective parameter in each water sample, $S_{i}$ the drinking water standard (mg/L) as per international guidelines (WHO, 2017). Fourth, sub-index of the ith parameter (Sli) was calculated for each parameter using Eq. (5):

(5) $$Sli=W_{i}\times q_{i},$$

where Sli is the sub-index of ith parameter. Finally, sum of the sub-index of all analysed parameters was estimated using Eq. (6):

(6) $$WQI=\sum_{i=1}^{n}Sli\text{.}$$

The WQI estimation in the present study was based on 8 different parameters, which are presented in Table 2 together with their assigned and relative weights. The WQI are classified into five categories (Sener et al., 2017): Excellent (<50); good (50–100); poor (100–200); very poor (200–300) and unfit for drinking purpose (>300).

#### 2.4.3 Pollution indices

Three pollution indices were used in order to assess the metal pollution. All three methods use specific equations in order to calculate the influence of metals. The MPI, Cd, and MEI are often used and very successful assessment methodologies for assessing metal pollution levels in mining activities (Bhuiyan et al., 2010a, b).

The ***metal pollution index (MPI)*** represents an efficient tool in indicating the quality status of waters, regarding the metal content, and possibly the pollution level. This method uses metal concentrations to measure pollution levels and drinking water quality (Prasad and Kumary, 2008). The method assigns a weight or a weighting between 0 and 1 and denoted ($W_{i}$) to each metal (Mohan et al., 1996). The parameters ($S_{i}$) and ($l_{i}$) represent the highest allowable and desirable standard values of each metal, respectively. These values were taken from the WHO (2011). The MPI was determined using Eq. (7) below (Mohan et al., 1996):

(7) $$MPI=\frac{\sum_{i=1}^{n}W_{i}Q_{i}}{\sum_{i=1}^{n}W_{i}}\text{,}$$

where $W_{i}$ is the unit weight, $Q_{i}$ is the sub-index of the i parameter, and n is the number of considered parameters. The sub-index $Q_{i}$ was calculated using Eq. (8):

(8) $$Q_{i}=\sum_{i=1}^{n}\frac{C_{i}}{S_{i}}\times 100,$$

where $W_{i}$ is the unit weightage of the ith parameter or the reciprocal number of the maximum allowable concentration (MAC), $Q_{i}$ is a sub-index, $C_{i}$ is the measured value of metal and Si is the standard value of metal. The critical pollution index value of MPI is defined by Bhardwaj et al. (2017): Acceptable overall pollution level (<100) and unacceptable overall pollution level (>100).

The ***contamination degree (Cd)*** is used to quantify the contamination level with the metal. The Cd summarizes the coupled effects of quality parameters considered harmful to household water (Backman et al., 1997), and was determined using Eqs. (9) and (10):

(9) $$Cd=\sum_{i=1}^{n}C_{fi}\text{,}$$

where

(10) $$C_{fi}=\frac{C_{Ai}}{C_{Ni}}-1\text{,}$$

where $C_{fi}$ is the contamination factor for the i-th component, $C_{Ai}$ is the analytical value for the i-th component, and $C_{Ni}$ is the upper permissible concentration of the i-th component (N denotes the ‘normative value’) provided by WHO (2011). Cd may be classified into three categories (Backman et al., 1997; Edet and Offiong, 2002) as follows: Low pollution (Cd<1); medium pollution (Cd1−3) and high pollution (Cd>3).

The ***metal evaluation index (MEI)*** is the study's last technique for assessing metal pollution in this surface water. It gives more accurate estimations by indicating the level of metal contamination in water (Edet and Offiong, 2002). The mean values of metals were used as the standard reclassification in this procedure, which was done using Eq. (11):

(11) $$MEI=\sum_{i=1}^{n}\frac{H_{c}}{H_{mac}}\text{,}$$

where $H_{c}$ and $H_{mac}$ are the monitored value and maximum admissible concentration (MAC) of the i-th parameter, respectively. According to Edet and Offiong (2002), the MEI are divided into 3 pollution classes: Low pollution (MEI <5); medium pollution (MEI 5–20) and high pollution (MEI > 20).

#### 2.4.4 Geochemical modeling

The significance of mineralogical phases in governing geochemical processes in surface water can be understood through geochemical modeling and the estimation of water saturation index. The validation of WSI results depend upon input data accuracy, degree of understanding of local geochemical processes, surface water system conceptualization and basic hydrochemical concepts. Changes in saturation state are useful for distinguishing the different stages of hydrochemical evolution and the identification of important geochemical reactions controlling de water chemistry (Parkhurst and Appelo, 1999). WSI were carried out using PHREEQC computer program. It operates according to the mass balance method, with determines the change in the chemical properties of the mineral species present in the water. WSI was calculated using Eq. (12).

(12) $$WSI=\mathrm{Log}\frac{IAP}{{Kt}_{sp\left(T\right)}}\text{,}$$

where IAP is ion activity product and ${Kt}_{sp\left(T\right)}$ is equilibrium solubility product of mineral. When WSI = 0; the water is in equilibrium with the mineral; WSI <0; the water is undersaturated, and capable of dissolving the mineral and WSI >0, the water is supersaturated, and capable of precipitating the mineral.

### 2.5 Multivariate statistical analysis

The values of the studied parameters were statistically analysed and compared to the World Health Organization, 2011, World Health Organization, 2017. Multivariate statistical analysis (MSA) is a sophisticated statistical analysis technique that allows large databases to be better interpreted. The MSA provides for a significant reduction in the size of a huge group of data while still preserving their relationships and similarities. The MSA was carried out in this study to determine the Pearson’s correlation matrix (with a significant level of P<0.05), principal component analysis (PCA), and cluster analysis (CA) by using the STATISTICA software.

The empirical association between two variables was determined using Pearson's correlation matrix (often referred to as X, Y). R stands for the correlation coefficient and is defined in the range [–1; +1]. Between 0.7 and 1, there is a strong linear correlation; between 0.5 and 0.7, there is a moderate linear association; between 0.3 and 0.5, there is a weak linear association; and between 0 and 0.3, there is little or no linear association (Armcanz and Anzecc, 2000). The correlation coefficient, $R_{xy}$ between two variables x and y with mean values $\bar{x}$ and $\bar{y}$ was determined using Eq. (13):

(13) $$R_{xy}=\frac{\sum_{i=1}^{n}\left(x_{i}-\bar{x}\right)\left(y_{i}-\bar{y}\right)}{\sqrt{\sum_{i=1}^{n}{\left(x_{i}-\bar{x}\right)}^{2}{\left(y_{i}-\bar{y}\right)}^{2}}}\text{.}$$

The PCA reduces the data and explains most of their variance. In general, PCA finds the direction in the multivariate space, which contains the maximum variability (Reimann et al., 2008).

The CA is a multivariate analysis approach. It is used to group parameters or samples in dendrograms that have comparable or similar properties and high linear association (Bhat et al., 2014).

### 2.6 Geostatistical modeling

The predictive maps of metals (Fe, Pb, As, Cd and Hg) and pollution indices (MPI, Cd and MEI) in surface water based on their mean values were generated by using ordinary kriging interpolation. It was used to predict the spatial distribution of metals and pollution indices in the studied section of the Lom River. It predicts unknown sites using measured data and a semivariogram model (Li et al., 2017). The geostatistical modeling was performed using ARCGIS 10.5 software (ESRI, 2016).

## 3 Results and discussion

The univariate statistical analysis of the physico-chemical parameters, physical parameters, major ions and metals and the comparison with the World Health Organization, 2011, World Health Organization, 2017 are reported in Table 1.

**Table 1:** Univariate statistical analysis of the studied parameters and comparison of their values with the World Health Organization, 2011, World Health Organization, 2017.

Parameters	Unit	Dry season				Rainy season				TM	WHO standards	
		Min	Mean	Max	SD	Min	Mean	Max	SD			
											2011	2017
**pH**		5.37	6.2	6.8	0.4	5.3	6.3	6.9	0.4	6.3	6.5–8.5	6.5–8.5
**EC**	μS/cm	27	40.5	48	5.8	28	41.6	101	14.5	41.1	-	-
**TDS**	mg/L	15	23.3	48	6.5	19	24.9	69	9.7	22.8	1000	-
**T°**	°C	20	25.1	29.5	2.5	19	25.3	31	4.2	25.2	-	-
**Alk**	mg/L	88.3	270.9	610	110.4	0.24	569.7	2341	110.4	420.3	-	-
**Tu**	NTU	126	234.4	510	85.9	117	172.2	310	38.2	203.3	5	4
**TSS**	mg/L	47.6	172.6	471	111.3	22.89	36.9	57.68	9.3	104.8	-	-
**Ca**^**2+**^	mg/L	0.17	1.9	2.86	0.9	1.34	2.1	2.77	0.5	2	-	-
**Mg**^**2+**^	mg/L	0.07	0.9	1.54	0.4	0.66	1.1	2.26	0.3	1.05	-	-
**Na**^**+**^	mg/L	0.13	2.4	5.97	0.5	0.01	2.7	11.40	1.9	2.6	200	-
**K**^**+**^	mg/L	0.11	1.05	2.40	0.5	1.13	1.6	5.77	0.9	1.3	-	-
**NH**_**4**_^**+**^	mg/L	0.001	0.07	0.60	0.1	0.01	0.2	4.14	0.8	0.1	-	-
**F**^**-**^	mg/L	0.01	0.07	0.70	0.1	0.01	0.08	0.12	0.03	0.08	1.5	1.5
**Cl**^**-**^	mg/L	0.09	0.2	0.88	0.2	0.27	1.6	20	4.1	0.9	200	-
**NO**_**3**_^**-**^	mg/L	0.01	0.2	1.14	0.2	0.05	0.8	6.04	1.1	0.5	50	50
**PO**_**4**_^**3-**^	mg/L	0	0.02	0.41	0.08	0	0.02	0.19	0.04	0.02	-	-
**SO**_**4**_^**2-**^	mg/L	0.27	0.6	2	0.4	0.28	1.1	3.62	0.8	0.9	200	-
**HCO**_**3**_^**-**^	mg/L	5.39	16.5	37.21	6.7	2.40	17.8	65.12	11.01	17.2	500	-
**Fe**	mg/L	0.17	0.7	1.86	0.5	2.12	2.7	3.07	0.3	1.7	0.3	-
**Pb**	mg/L	0.04	0.5	2.78	0.7	0.002	0.005	0.008	0.001	0.2	0.01	0.01
**As**	mg/L	0.013	0.3	1.43	0.5	0.001	0.005	0.009	0.002	0.2	0.01	0.01
**Cd**	mg/L	0.001	0.004	0.007	0.001	0.0001	0.0005	0.0008	0.0002	0.002	0.003	0.003
**Hg**	mg/L	0.001	0.005	0.021	0.005	0.001	0.004	0.007	0.002	0.005	0.006	0.006

**Legend:** TM = total mean value; SD = Standard deviation; WHO = World Health Organization.

**Table 2:** Water quality index (WQI).

Parameters	Unit	Mean concentration ($C_{i}$)	WHO (2017) ($S_{i})$	Weight ($w_{i})$	Relative weight ($W_{i})$	Grade quality (qi)	Sub-index (${Sl}_{i}$)
**pH**		6.3	6.5–8.5	4	0.108	83.6	9
**Tu**	NTU	203.3	4	3	0.081	5083	412.1
**F**^**-**^	mg/L	0.08	1.5	5	0.135	5.3	0.7
**NO**_**3**_^**-**^	mg/L	0.5	50	5	0.135	1	0.14
**Pb**	mg/L	0.2	0.01	5	0.135	2400	324.3
**As**	mg/L	0.2	0.01	5	0.135	1700	229.7
**Cd**	mg/L	0.002	0.003	5	0.135	80	10.8
**Hg**	mg/L	0.004	0.006	5	0.135	78.3	10.6
**TOTAL**				37	1	83.6	9
**WQI**		997.5					

### 3.1 Variation of physico-chemical and physical parameters

**Figure 4:**
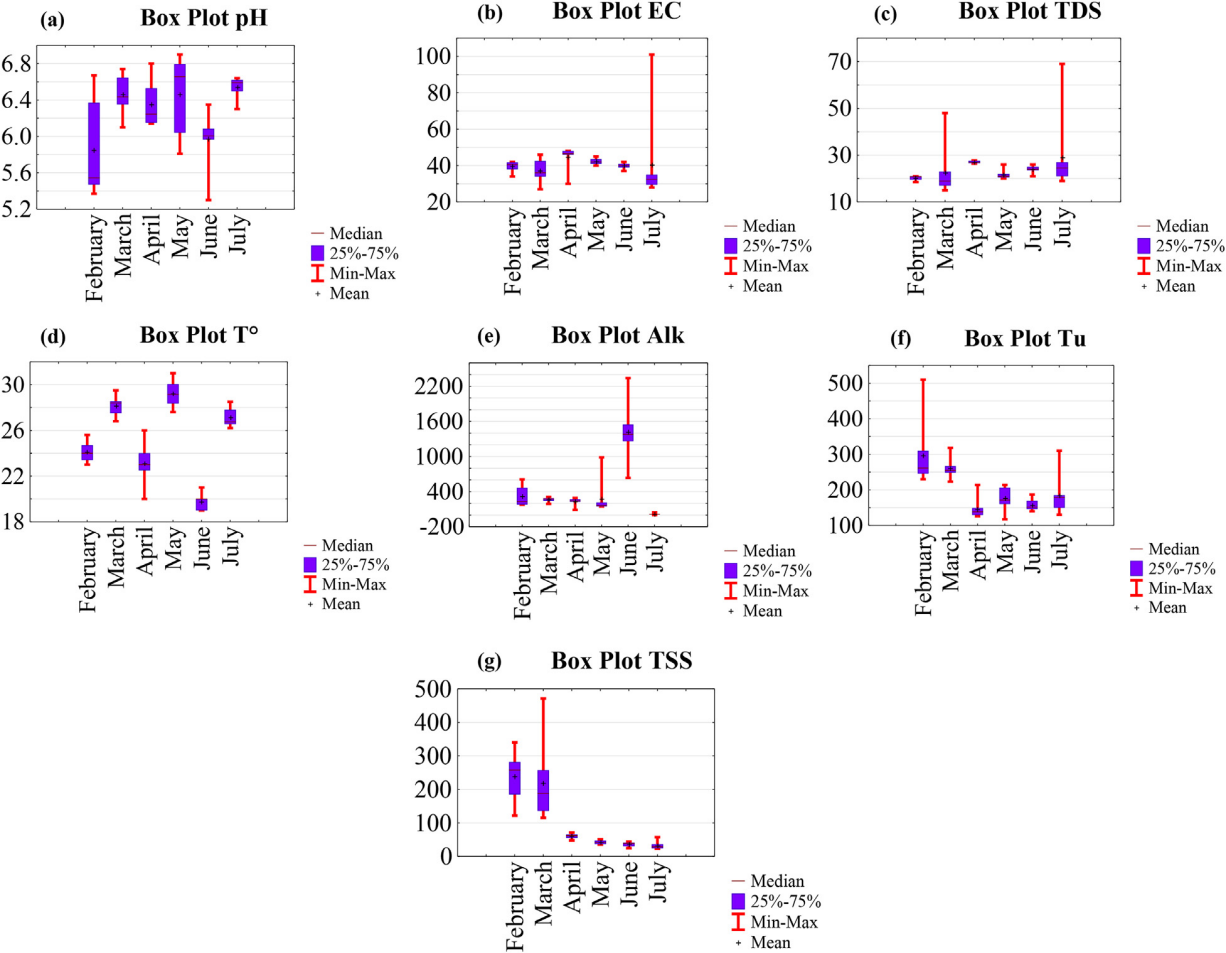
Box plots of physico-chemical and physical parameters: (a) pH; (b) EC; (c) TDS and (d) T° analysed in situ and (e) Alk, (f) Tu and (g) TSS analysed in laboratory.

The variation range of the main physico-chemical parameters (pH, EC, TDS and T°) measured in situ and physico-chemical parameters (Alk, Tu and TSS) determined in laboratory are presented in the box plots in Figure 4.

The Lom River draining the gold mining site at Gankombol was characterized by acidic to near-neutral pH in dry season (5.37–6.8) and in rainy season (5.3–6.9) (Table 1 and Figure 4a). The lowest pH values in the Lom River might be attributed to the weathering of sulphide minerals. Sulphide minerals such as pyrite, arsenorpyrite, and galena which oxidize when exposed to water, are commonly found with mineral paragenesis of gold in the East and Adamawa region of Cameroon (Suh et al., 2006; Vishiti et al., 2019; Ndonfack et al., 2021). The pH drops as a result of this oxidation. The pH values found in this study were similar to those obtained in investigations conducted at other well-known gold mining locations (Hakkou et al., 2005; Attua et al., 2014; Rakontontrabe et al., 2017b; Mambou et al., 2020a, b; Ayiwouo et al., 2020, 2021) and glacial lakes from Rodnei mountains in Romania (Roșca et al., 2020).

The EC were relatively low and ranged from 27-48 μS/cm in dry season and 28–101 μS/cm in rainy season (Table 1 and Figure 4b). These low conductivities were similar to those seen in most waters in eastern Cameroon and Colombia gold mining zones (Rakontondrabe et al., 2017a; Mambou et al., 2020a, b; Corredor et al., 2021). They were lower than the EC of some bottles waters from Roumania (Dippong et al., 2020).

The TDS values were also relatively low (Table 1 and Figure 4c) and lower than the limit of 1000 mg/L set by the WHO standard (2011) (see Table 1). The total mean value of TDS (23.3 mg/L) was lower than the mean value of 24.2 mg/L found in the Djengou river in Batouri's gold district (Mambou et al., 2020b) and the mean value of 9496 mg/L found in Ghana's Akyem Abuakwa gold district (Attua et al., 2014).

The temperature of samples ranged from 20 to 29.5 °C in dry season and 19–31 °C in rainy season (Table 1 and Figure 4d). The total mean value was 25.2 °C.

The Alk ranged from 88.3 to 610 mg/L in dry season and 0.24–2341 mg/L in rainy season (Table 1 and Figure 4e). 77% of the water samples were lower than the total mean value of 420.3 mg/L. The Alk concentrations were relatively high and increased in rainy season due to the increased leaching by runoff water of soils rich in organic matter. The total mean alkalinity of 420.3 mg/L was similar to the mean value of 427.3 mg/L found in the gold district of Bétare-Oya-Cameroon (Rakontondrabe et al., 2017a). It was higher than those obtained in streams associated with artisanal gold mining in Colombia (Corredor et al., 2021).

The Lom River draining the gold mining site was turbid (117–510 NTU) (Table 1 and Figure 4f). Tu values exceeded the limits of 5 and 4 NTU set by the World Health Organization, 2011, World Health Organization, 2017. 43.7% of the water samples had a turbidity higher than the total mean value (203.3 NTU). W6 (March) and W1 (July) had the highest and lowest values, respectively (Figure 4f). The Tu values decreased in rainy season with increasing of precipitation, here reflecting the impact of the seasons.

TSS ranged from 47.6 to 471 mg/L in dry season and 22.89–57.68 mg/L in rainy season (Table 1 and Figure 4g). As turbidity, the TSS decreased with the increase in precipitation. It is also important to note that during the rainy season, gold mining was momentarily stopped due to heavy rains. The high concentrations of TSS caused by excavation works, deforestation, dredging of beds, rivers and panning of gold were decreasing considerably. The highest values were found in the samples in the gold mining site. Whereas the lowest values were found in samples taken upstream and downstream of the mining site, demonstrating the spatial influence of mining extraction. The total mean values of Tu and TSS (Table 1) were higher than those obtained in some bottles waters from Roumania (Dippong et al., 2020) and glacial lakes from Rodnei mountains in Romania (Roșca et al., 2020). They were similar to those observed in certain gold mining sites from Cameroon (Rakontontrabe et al., 2017a; Mambou et al., 2020a, 2020b; Ayiwouo et al., 2020, 2021) and in rivers in the forest/savanna buffer zone, such as the Sanaga and the Mbam (Ndam et al., 2016).

### 3.2 Variation and source of major ions

**Figure 5:**
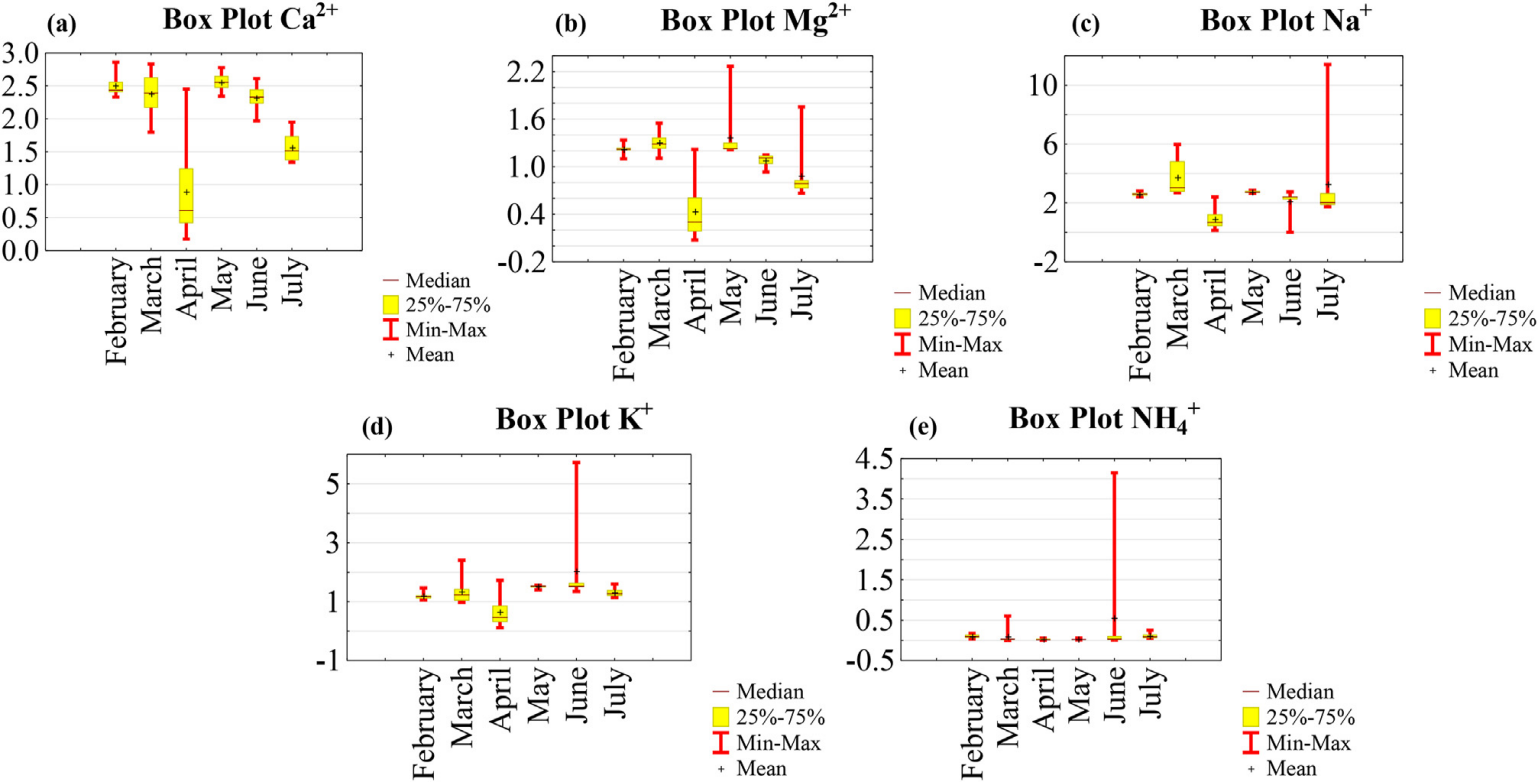
Box plots of major cations: (a) Ca^2+^; (b) Mg^2+^; (c) Na^+^; (d) K^+^ and (e) NH_4_^+^.

**Figure 6:**
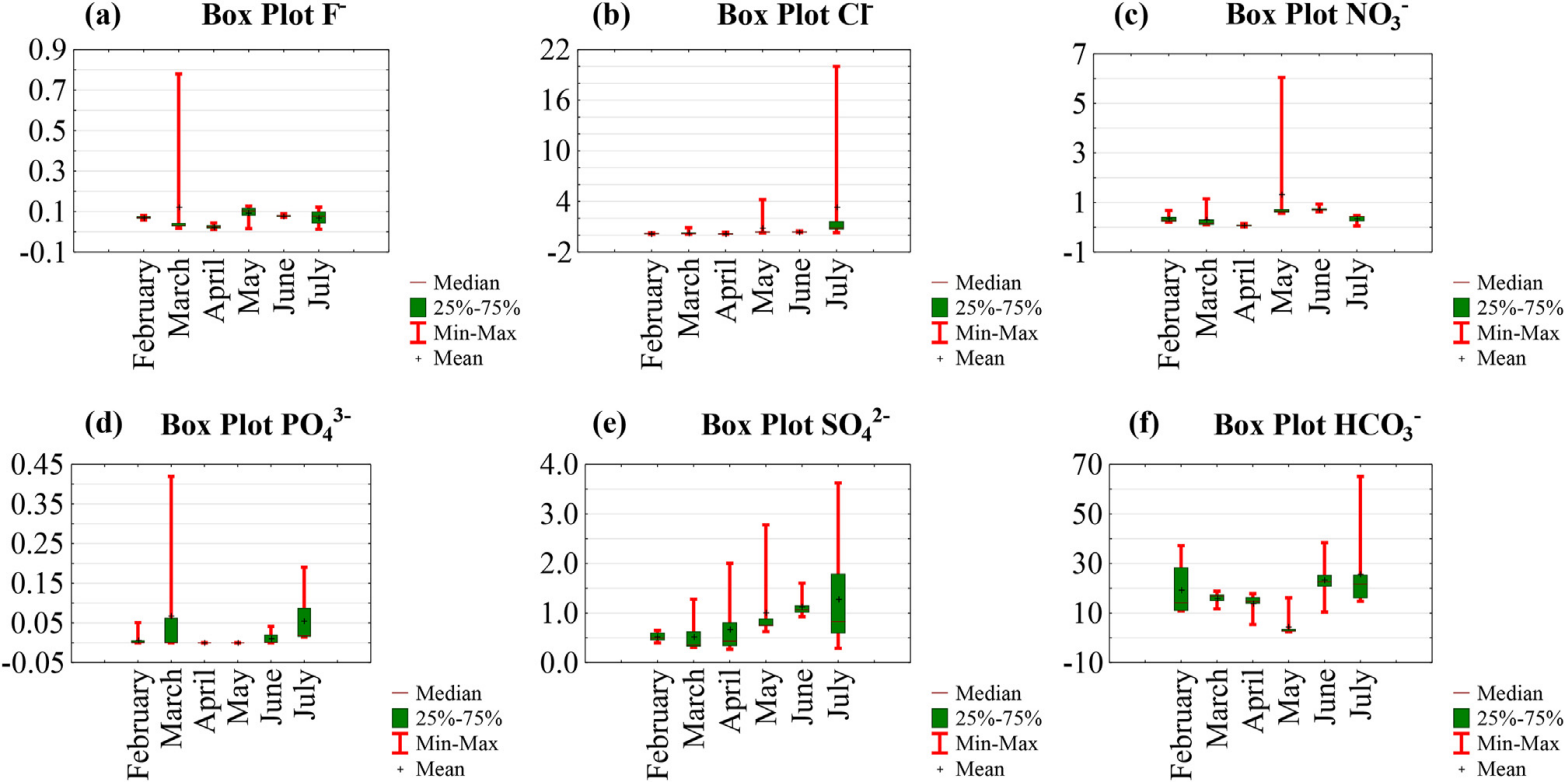
Box plots of major anions: (a) F^−^, (b) Cl; (c) NO_3_; (d) PO_4_^3^; (e) SO_4_^2^ and (f) HCO_3_^-^.

The variation range of major ions is illustrated in the box plots in Figures 5 and 6.

The major ion concentrations decreased in the following order in dry season: HCO_3_^-^ > Na^+^ > Ca^2+^ > K^+^ >Mg^2+^ > SO_4_^2-^ > NO_3_^-^ > Cl^−^ > F^−^ > NH_4_^+^ > PO_4_^3-^; in rainy season: HCO_3_^-^ > Na^+^ > Ca^2+^ > K^+^ > Cl^−^ > SO_4_^2-^ > Mg^2+^ > NO_3_^-^ > NH_4_^+^ > F^−^ > PO_4_^3-^ and globally:: HCO_3_^-^ > Na^+^ > Ca^2+^ > K^+^ >Mg^2+^ > Cl^−^ > SO_4_^2-^ > NO_3_^-^ > NH_4_^+^ > F^−^ > PO_4_^3-^.

The concentrations of major cations (Na^+^, Ca^2+^, Mg^2+^, K^+^ and NH_4_^+^) were relatively low in dry season and in rainy season (Table 1 and Figures 5a, 5b, 5c, 5d and 5e). 35.4%, 35.4%, 3.1%, 52.1% and 87.5% of the water samples failing below the total mean concentration of Na^+^, Ca^2+^, Mg^2+^, K^+^ and NH_4_^+^, respectively. The concentrations of Na^+^ were below the WHO standard (2011).

The concentrations of major anions were also relatively low during the two seasons, with the exception of HCO_3_^-^ which was the dominant major ion in these waters (Table 1 and Figures 6a, 6b, 6c, 6d, 6e and 6f). Chlorides were more abundant than sulfates (Table 1). 68.7%, 87.5%, 62.5%, 83.3% and 58.3% of the water samples had a concentration below the total mean concentration of Cl^−^, F^−^, NO_3_^-^, PO_4_^3-^, SO_4_^2^ and HCO_3_^-^, respectively. The concentrations of HCO_3_^-^ increased in dry season and all the concentrations of major ions were below the World Health Organization, 2011, World Health Organization, 2017.

The anions of the Lom River in the gold mine at Gamkombol were mainly dominated by the HCO_3_^-^ and the cations by Na^+^ and Ca^2+^ (Table 1 and Figures 5 and 6). HCO_3_^-^ concentrations might derive from the formation of carbonic acid and its dissociation. These concentrations were elevated in rainy season due to the increased leaching of organic-rich soils by runoff from rainwater. Na^+^ and K^+^ concentrations in these waters would be due to the weathering of evaporite, silicate and quartzo-felspathic vein present at Gankombol (see Figure 2b). The presence of Ca^2+^ and Mg^2+^ in these waters results from the weathering silicate or granite rich in amphibole and biotite minerals and quartzo-felspathic vein (see Figure 2b). Ca^2+^ and Mg^2+^ are responsible for the hardness of water (Li et al., 2014). Cl^−^ in these waters would come from the weathering of rock salt and evaporate minerals. The presence of SO_4_^2-^ in this surface water could be attributed to the dissolution of evaporite or the surrounding granite rocks (see Figure 2b). The natural source of NO_3_^-^ is atmospheric deposition of nitrate salts (Singh et al., 2008). NH_4_^+^ is the main ion that contributes to the eutrophication of water. Its presence in these waters might be attributed to the decomposition of organic waste matter in water. Usually, F^−^ originates from weathering of sedimentary and igneous rocks. At Gankombol, the presence of F^−^ in waters would come from the alteration of the surrounding granite and granodiorite rocks (see Figure 2b).

In conclusion, except HCO_3_^-^, the concentrations of other major ions follow the same trends with major ion concentrations determined in the gold districts of Batouri (Mambou et al., 2020b) and Bétare-Oya (Rakontondrabe et al., 2017b).

### 3.3 Variation and source of metals

**Figure 7:**
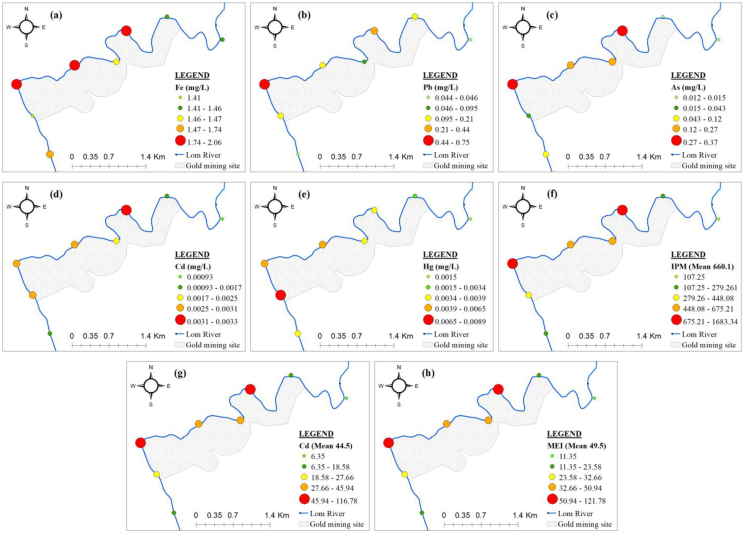
Spatial variation of metals and pollution indices: (a) Fe; (b) Pb; (c) As; (d) Cd; (e) Hg; (f) metal pollution index (MPI); (g) contamination degree (CD) and (h) metal evaluation index (MEI).

The spatial variation of metals and pollution indices (MPI, CD and MEI) based on their mean values is presented in Figure 7.

The metal concentrations decreased in the following order: Fe > Pb > As > Hg > Cd. The concentrations of metals ranged from low to significant in dry season and in rainy season (Table 1 and Figure 7). For Fe, Pb, As, Cd and Hg, 46.9%, 78.1%, 84.3%, 53.1% and 50% of the water samples had a concentration lower than the total mean value (Table 1), respectively. 90.6%, 80%, 50%, 53% and 25% exceeded the limits set by the World Health Organization, 2011, World Health Organization, 2017, respectively. The highest concentrations of Fe, As and Cd were found in the gold mining site's sampling point W3 (Figures 7a, 7c and 7d). The highest concentrations of Pb and Hg in the sampling point W6 (Figures 7b and 7e). Except for Pb, where the lowest concentrations were found in the sampling point downstream the mining site W6 (Figure 7b), the lowest concentrations of other metals were found in the sampling point upstream the gold mining site (W1) (Figures 7a, 7c, 7d and 7e).

#### 3.3.1 Iron (Fe)

The presence of Fe concentrations in this surface water could come from the weathering of pyrite (satellite minerals associated with gold), tourmaline (Fe-rich) and tourmaline (Fe-rich)-wolframite bearing quartz veins and surrounding granite and gneiss rocks rich in biotite, amphibole and chlorite minerals (see Figure 2b). Fe could also come from the leaching of the ferralitic soils which cover all the countries south of the 8th parallel (Segalen, 1967). Fe concentrations in West and Center African rivers are often high due to leaching from lateritic-ferralitic soils (Hernandez, 2003). The minimum concentration of Fe was recorded upstream of the gold mining site (Figure 7a), which could reflect the spatial influence of mining activities on the Lom River. The Fe concentrations obtained in this study were similar to those found in Cameroon's gold mining districts (Rakontodrabe et al., 2017a; Mambou et al., 2020a, b; Ayiwouo et al., 2020) and lower than those obtained in the glacial lakes from Rodnei mountains in Romania (Roș;ca et al., 2020).

#### 3.3.2 Lead (Pb)

The presence of Pb in these waters is likely related to the weathering of galena (PbS). Gold in the study area is orogenic and the main mineral paragenesis consist of galena and others minerals such as pyrite, chalcopyrite, sphalerite and arsenopyrite (Suh et al., 2006; Vishiti et al., 2019; Ndonfack et al., 2021). Pb could also come through miners' excavation activity, which results in the alteration and leaching of metal from waste rock storage sites or mining gear exhaust pipes (Aranguren, 2008). The total mean concentration of Pb (0.2 mg/L) was higher than the mean concentration obtained in the gold districts of Bonikro (Bamba, 2012) and Bétare-Oya (Rakontondrabe et al., 2017a; Mambou et al., 2020a).

#### 3.3.3 Arsenic (As)

The high concentrations of As in the Lom River may be due to the weathering of arsenopyrite, a gold-related satellite mineral. Arsenic is obtained naturally from rocks and gold ore, such as arsenopyrite (Asamoah, 2012). The gold in this area is characterized as orogenic gold, with arsenopyrite being the major mineral paragenesis (Suh et al., 2006; Vishiti et al., 2019; Ndonfack et al., 2021). Arsenic (As) could also come from the weathering of metasedimentary rocks, clay minerals and atmospheric precipitation from incineration. The total mean concentration of As (0.2 mg/L) was higher than that found in the gold mining districts of Bétare-Oya (Rakotondrabe et al., 2017a) and Batouri (Mambou et al., 2020b), respectively. This mean value was similar to that found in the gold mining site of Wakaso (Ayiwouo et al., 2021) and lower than that obtained in the glacial lakes from Rodnei mountains in Romania (Roșca et al., 2020).

#### 3.3.4 Cadmium (Cd)

The main natural source of Cd is sphalerite. It is present in most of the gold veins in Cameroon (Suh et al., 2006; Vishiti et al., 2019; Ndonfack et al., 2021). Therefore, the presence of Cd in this surface water is thought to be due to the water-rock interaction. The total mean concentration of Cd (0.002 mg/L) was lower than that found in the Lom River in the Wakaso gold mining site (Ayiwouo et al., 2021) and the glacial lakes from Rodnei mountains in Romania (Roș;ca et al., 2020). The obtained Cd concentrations were similar to those found in the gold areas of Bétare-Oya (Rakotondrable et al., 2017a) and Batouri (Mambou et al., 2020b).

#### 3.3.5 Mercury (Hg)

The presence of Hg in the Lom River could be due to bushfires or the gold recovery processing of gold with mercury. During the washing of gold by artisanal miners, mercury particles were found in the pan. In addition to this, the mining company heats mercury with gold during processing for gold recovery. So when it comes into contact with gold, mercury vapors can settle in the water and contaminate it. The health risk is very high for these miners and even local residents who regularly inhale this highly toxic substance. Given the very high toxicity of mercury, Cameroon's mining legislation (Mining code of Cameroon, 2016) prohibits its use in mining sites. The effects of Hg on health are an accumulation in the muscles, the liver and damage to DNA (Fernandez -Luqueno et al., 2013). The total mean concentration of Hg (0.005 mg/L) was higher than to that found in Batouri's gold district (Mambou et al., 2020b) but lower than that found in the Lom River at Wakaso gold mining site (Ayiwouo et al., 2021).

#### 3.3.5 Influence of seasons on the variation of metals in the lom river

The concentrations of Pb, As, Cd and Hg were higher during the dry season than the rainy season due to the intensification of gold mining activities. Gold mining in Gankombol is mainly done during the dry season due to lack of rain and easy access to the mining site. In addition, during the dry season, there was an increase in metal concentrations in the Lom River due to the reduction in water volume and flow. Metal concentrations also increasing due to evaporation from water bodies (Edokpayi et al., 2016). In rainy season, gold mining activities were less intense. The decrease in Pb, As, Cd and Hg concentrations was mainly due to the dilution of the Lom River by rainwater. Unlike other metals, Fe concentrations increase during the rainy season due to mine runoff, leaching of lateritic soils by rainwater, and weathering of pyrite, tourmaline (rich in Fe) and tourmaline (rich in Fe-wolframite) and the surrounding granitic and gneissic rocks rich in biotite, amphibole and chlorite observed at Gankombol (see Figure 2b).

### 3.4 Water quality and geochemical modeling

#### 3.4.1 Hydrochemical signatures and water quality suitability of irrigation purpose

**Figure 8:**
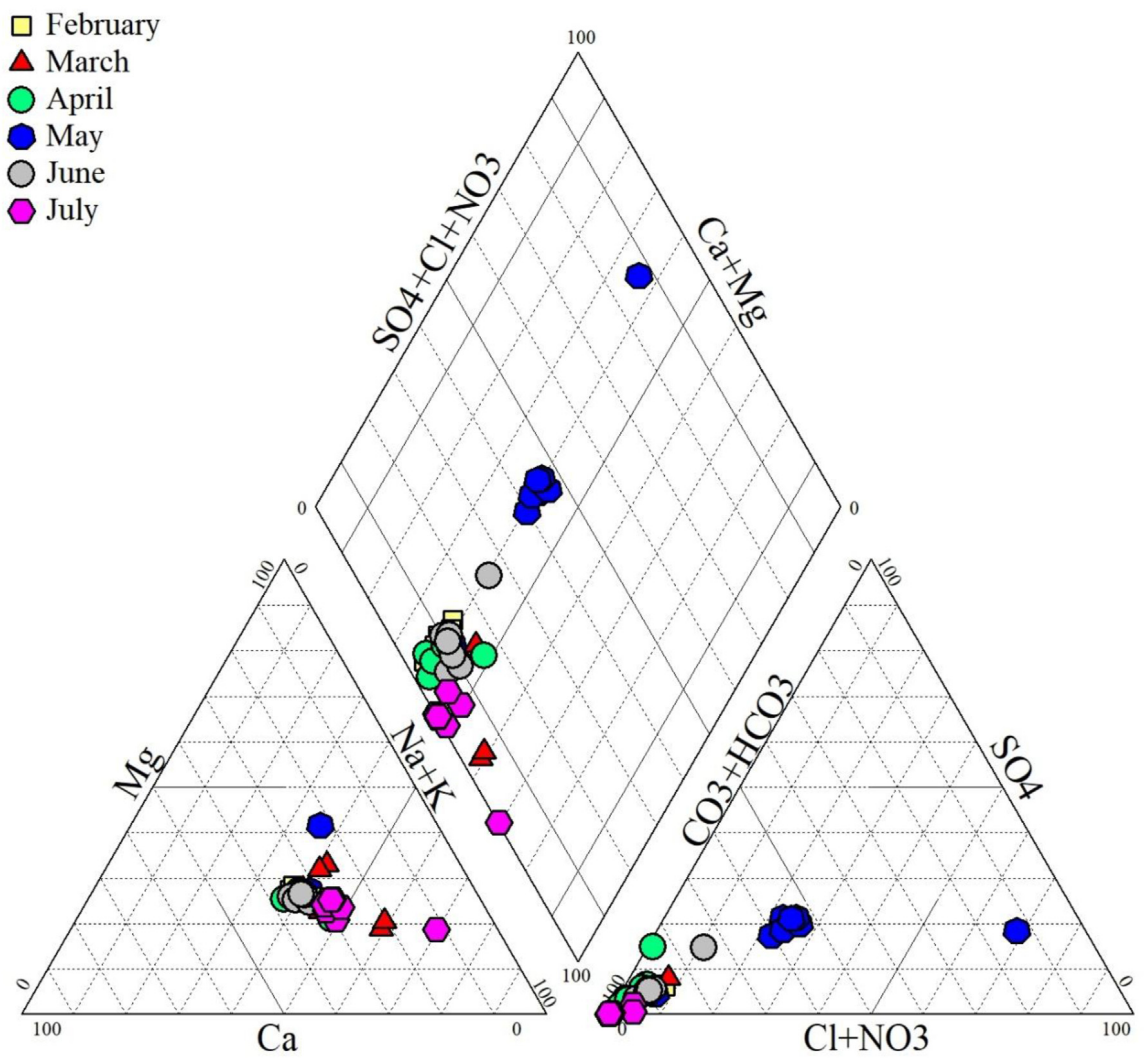
Piper’s diagram.

The water types were Ca–Mg–HCO_3_ and Ca–Mg–Cl–SO_4_ (Figure 8). The main water type was alkaline earth-magnesium-bicarbonate (Ca–Mg–HCO_3_) which represented 97.9% of water samples (Figure 8). The second type was alkaline earth-magnesium-chloride-sulfate (Ca–Mg–Cl–SO_4_) and accounts for 2.1% of water samples. The Lom River was alkaline earth-magnesium-bicarbonate (Ca–Mg–HCO_3_) without dominant cations with high levels of bicarbonate (HCO_3_). The same results were found in the Lom River in the localities of Bétare-Oya (Rakondrabe et al., 2017a), Wakaso (Ayiwouo et al., 2021) and in the Awash River in Ethiopia (Yitbarek et al., 2012).

**Figure 9:**
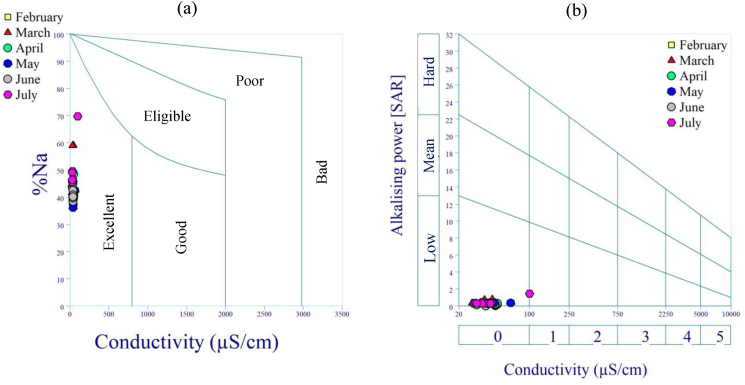
(a) Wilcox diagram and (b) Riverside diagram.

All the samples were excellent for irrigation (Figure 9a). The alkalizing power (SAR) ranged from 0.07 to 1.5, while the conductivity ranged from 20 to 100 μS/cm (Figure 9b). The analysed stretch of the Lom River was suitable for irrigation based on the percentages of sodium and conductivity obtained on the Wilcox diagram (Figure 9a). The alkalizing power of the samples was minimal, indicating that the surface water was of acceptable quality (Figure 9b). This surface water can be applied to a variety of soil types.

#### 3.4.2 Water quality index (WQI)

The WQI is a valuable and unique assessment to describe the overall state of water quality in a single term. it is useful for the selection of the appropriate treatment technique and for addressing the issues involved. Information on water quality is important to the public and to legislative decision-makers. According to Table 2, the mean WQI (997.5) indicated that the Lom River at Gankombol was unsuitable for drinking purpose (WQI >300). The mean WQI (997.5) obtained was lower than the mean score of 18,856.1 found in natural waters from the Ameka metallogenic district in Southeastern Nigeria (Egbueri and Enyigwe, 2020). The WQI values were higher than those obtained in the glacial lakes from Rodnei mountains in Romania (Roș;ca et al., 2020).

#### 3.4.3 Pollution indices

The values of pollution indices (MPI, CD and MEI) were very high at all the sampling point (Figures 7f, 7g and 7h). The highest values were observed at points W3 and W6 and the lowest value at point W1 (Figures 7f, 7g and 7h). The MPI values (107.25–1683.34) were higher than the critical pollution index (100). The Cd values (6.35–116.78) exceeded the high pollution class (Cd>3). In addition, the mean value of MEI (49.5) was higher than the high pollution class (MEI>20). The mean values of MPI, CD and MEI were higher than those obtained in the glacial lakes from Rodnei mountains and some bottles waters in Romania (Roșca et al., 2020; Dippong et al., 2020).

Regardless of the tree methods used for assessing metal pollution, the contamination levels were constantly exceeded the highest limit classes. These results suggested very high metal pollution of the Lom River at Gankombol.

#### 3.4.4 Geochemical modeling

**Figure 10:**
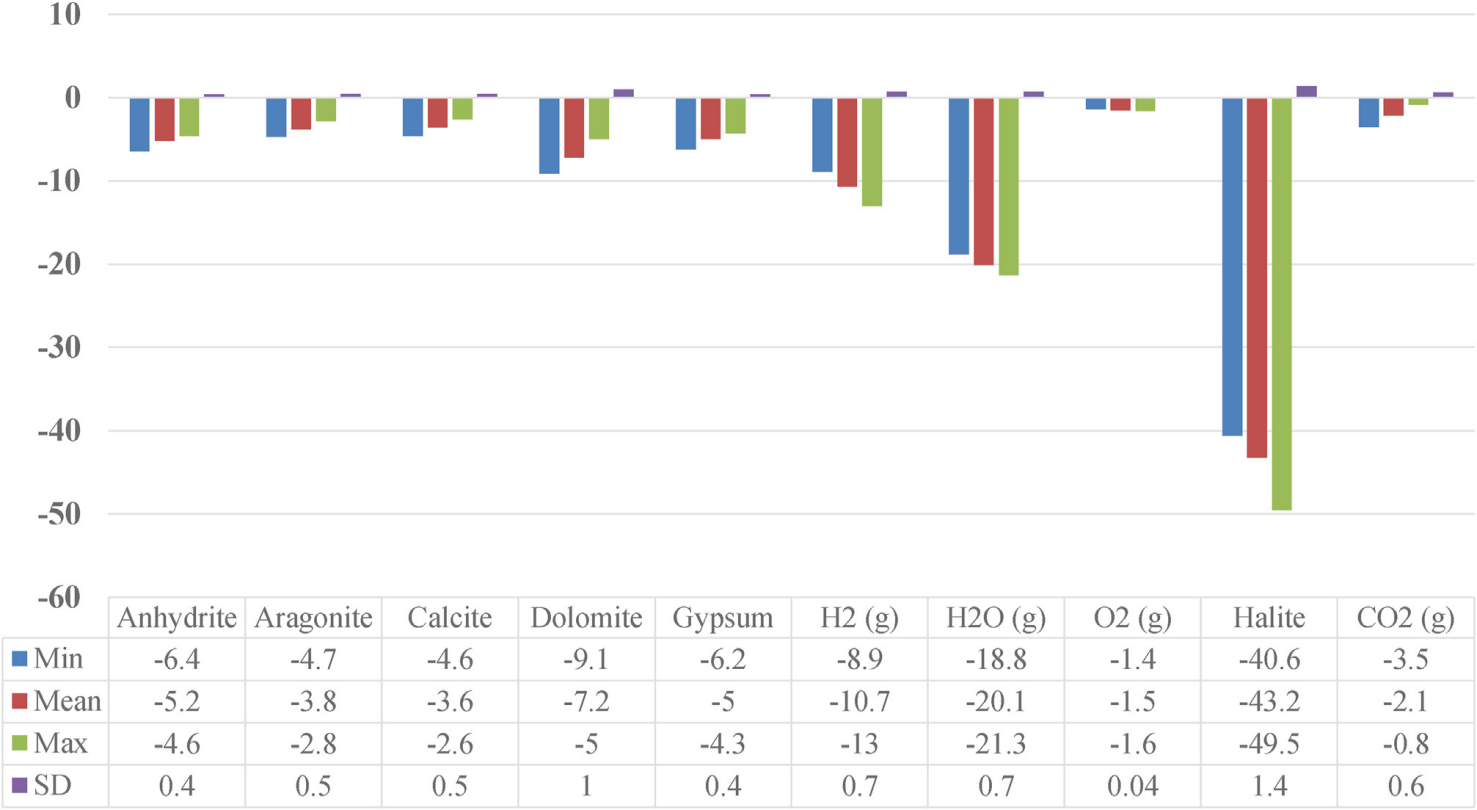
Variation and descriptive statistics of WSI (N = 48).

The variation and descriptive statistics of geochemical phases with the determination of the water saturation index (WSI) of all the samples are reported in Figure 10. The WSI were less than zero (WSI <0) for all the studied phases’ minerals (Figure 10). This indicates that the water was undersaturated, allowing the mineral to dissolve. The WSI was influenced by the area's lithology and climatological variables. In the auriferous district of Bétare-Oya (Rakontondrabe et al., 2017a), where climate and lithology are almost identical, similar findings were seen.

### 3.5 Multivariate statistical analysis

#### 3.5.1 Pearson correlation analysis

The linear correlation between the variables in waters was assessed using Pearson’s correlation matrix (Table 3). Strong linear correlations were found between: EC-TDS (R = 0.77), EC-Na^+^ (R = 0.78), TDS-Na^+^ (R = 0.76), Pb–As (R = 0.84), Cl^−^ –SO_4_^2-^ (R = 0.80) and TSS-Cd (R = 0.80). Moderate linear correlations between: Tu-Cd (R = 0.64), K^+^ -Hg (R = 0.61), Tu-TSS (R = 0.59), Mg^2+^-NO_3_^-^(R = 0.58), Ca^2+^-Mg^2+^ (R = 0.57), TSS-Pb (R = 0.51) and TSS-As (R = 0.52) (Table 3). A strong linear correlation existed between EC and TDS (R = 0.77) because they define the level of salinity in waters. The sources of TDS and EC would come from nature, geological conditions, seawater, and human activities (domestic and industrial wastes and agriculture) (Carreira et al., 2014; Rusydi et al., 2015). The strong correlation between TDS -Na^+^ (R = 0.76) explained the fact they indicate the natural processes defining the chemistry of surface waters. Strong linear correlation between EC-Na^+^ (R = 0.78) was also observed, both parameters which define the potential use of water for irrigation. Pb and As (R = 0.84) had strong linear correlation because the main gold paragenesis consist of galena and arsenopyrite. The strong correlation found between Cl^−^ –SO_4_^2-^ (R = 0.80) potentially indicated that they come from the dissolution of evaporite. Strong correlation between TSS and Cd (R = 0.80) suggested that TSS contained in these waters probably fixed the Cd^2+^ ions. Physical pollution, which is the results of excavation operations, dredging of the Lom River, and gold panning, were responsible for the moderate linear correlation between Tu and TSS (R = 0.59). A moderate linear correlation was also observed between Ca^2+^ and Mg^2+^ (R = 0.57), elements mainly responsible for the hardness of the water. TSS had moderate linear correlations with Pb (R = 0.51) and As (R = 0.52) suggesting that these metals may has been fixed onto very fine particles (<0.45 μm) and/or associated colloids. The moderate linear correlations between the major ions Mg^2+^ –NO_3_^-^ (R = 0.58) and NO_3_^-^ –SO_4_^2-^ (R = 0.52) would be due to the decomposition of organic waste matter in water and the weathering of surrounding rocks.

**Table 3:** Pearson’s correlation matrix.

Variables	pH	EC	TDS	T°	Tu	TSS	Alk	Ca^2+^	Mg^2+^	Na^+^	K^+^	NH_4_^+^	F^-^	Cl^-^	NO_3_^-^	PO_4_^3-^	SO_4_^2-^	HCO_3_^-^	Fe	Pb	As	Cd	Hg
**pH**	1.00																						
**EC**	0.01	1.00																					
**TDS**	0.15	**0.77**	1.00																				
**T°**	0.48	-0.01	0.04	1.00																			
**Tu**	-0.15	0.19	0.09	-0.48	1.00																		
**TSS**	-0.30	-0.14	-0.29	-0.46	**0.59**	1.00																	
**Alk**	-0.09	0.00	-0.22	-0.03	0.47	0.32	1.00																
**Ca**^**2+**^	-0.31	0.12	-0.35	-0.02	0.26	0.42	0.40	1.00															
**Mg**^**2+**^	0.04	0.45	0.22	0.20	0.18	0.16	0.24	**0.57**	1.00														
**Na**^**+**^	0.16	**0.78**	**0.76**	0.04	0.32	-0.02	-0.05	-0.01	0.42	1.00													
**K**^**+**^	0.25	0.22	0.06	0.36	-0.01	-0.19	-0.07	0.23	0.26	0.37	1.00												
**NH**_**4**_^**+**^	-0.11	0.03	0.38	-0.10	0.21	-0.09	-0.04	-0.30	0.08	0.19	-0.22	1.00											
**F**^**-**^	0.01	-0.13	-0.21	0.10	0.03	-0.10	0.07	0.23	-0.10	-0.05	0.15	-0.15	1.00										
**Cl**^**-**^	0.16	-0.18	0.01	0.01	-0.19	-0.24	-0.24	-0.26	-0.14	-0.11	0.02	0.04	0.05	1.00									
**NO**_**3**_^**-**^	0.17	-0.04	-0.11	0.33	-0.32	-0.25	-0.05	0.18	**0.58**	-0.10	0.19	-0.11	0.07	0.16	1.00								
**PO**_**4**_^**3-**^	0.24	-0.05	0.00	0.07	-0.05	-0.06	-0.11	-0.23	0.01	0.03	0.04	-0.04	-0.11	0.26	-0.12	1.00							
**SO**_**4**_^**2-**^	0.23	-0.25	-0.08	0.18	-0.35	-0.40	-0.29	-0.24	-0.01	-0.20	0.10	0.07	0.18	**0.80**	**0.52**	0.22	1.00						
**HCO**_**3**_^**-**^	0.06	0.46	0.71	-0.34	0.49	0.08	0.07	-0.45	-0.04	**0.58**	-0.14	0.35	-0.08	0.04	-0.30	0.06	-0.13	1.00					
**Fe**	0.33	0.16	0.32	0.33	-0.52	-0.70	-0.27	-0.41	-0.16	-0.07	0.17	-0.11	-0.09	0.30	0.28	-0.12	0.47	0.05	1.00				
**Pb**	-0.40	-0.10	-0.14	-0.49	0.31	**0.51**	0.34	0.18	0.05	-0.08	-0.22	-0.01	-0.07	-0.12	-0.13	-0.09	-0.21	0.22	-0.18	1.00			
**As**	-0.24	-0.09	-0.14	-0.64	0.46	**0.52**	0.35	0.18	0.04	-0.12	-0.29	0.02	-0.06	-0.13	-0.13	-0.14	-0.22	0.24	-0.18	**0.84**	1.00		
**Cd**	-0.16	-0.13	-0.30	-0.38	**0.64**	**0.80**	0.36	0.38	0.16	0.14	0.00	-0.08	0.03	-0.23	-0.27	0.01	-0.42	0.14	-0.71	**0.55**	**0.53**	1.00	
**Hg**	0.08	0.00	-0.04	-0.01	0.17	0.24	0.01	0.02	0.06	0.32	**0.61**	-0.10	-0.11	0.06	-0.11	0.12	-0.05	0.10	-0.20	-0.02	-0.15	0.41	1.00

Bold values indicate strong and moderate linear correlations.

#### 3.5.2 Principal components analysis (PCA)

**Figure 11:**
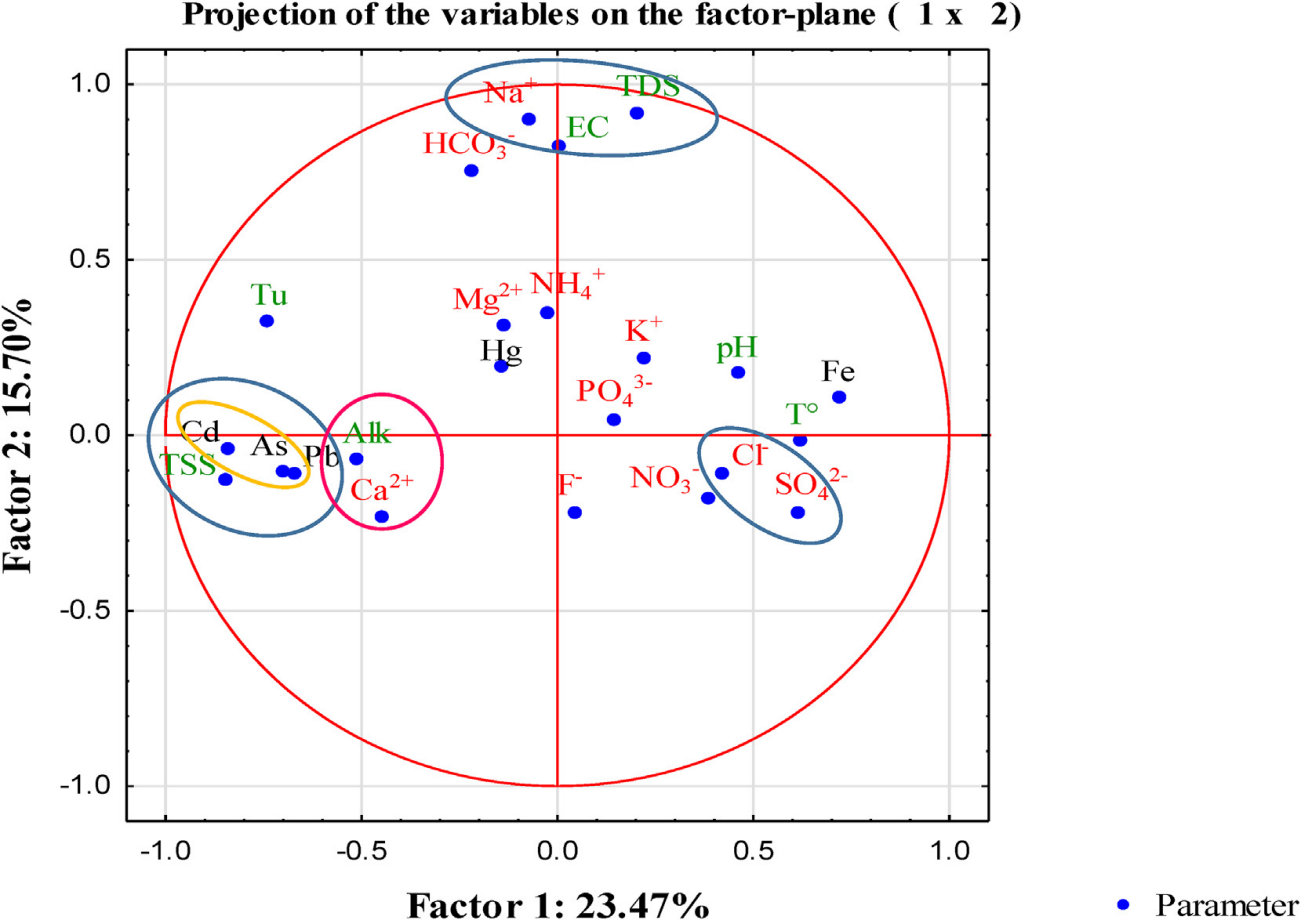
Principal component analysis.

In addition to the Pearson’s correlation analysis, PCA was used to identify the most significant parameters (components), their interrelationships, and the variability existing between them. The variables of PCA are distributed in the axes F1 – F2. Five groups are observed in Figure 11. The first group was composed of Na^+^, TDS and EC. They are the main parameters responsible for the processes defining the chemistry of the surface water and the suitability of the water for irrigation. The second group composed of TSS, Pb, As, and Cd confirmed that the these metal ions were fixed in the fine suspended matter contained in the Lom River. The third group composed of Pb, As and Cd could indicate that the paragenesis at Gankombol consist of galena, arsenopyrite and sphalerite. The fourth group was made up of Ca^2+^ and Alk, parameters which represented the alkalinity of water. The last group was made up of Cl^−^ and SO_4_^2-^ and suggested that these elements could come from the dissolution of evaporite.

#### 3.5.3 Cluster analysis (CA)

**Figure 12:**
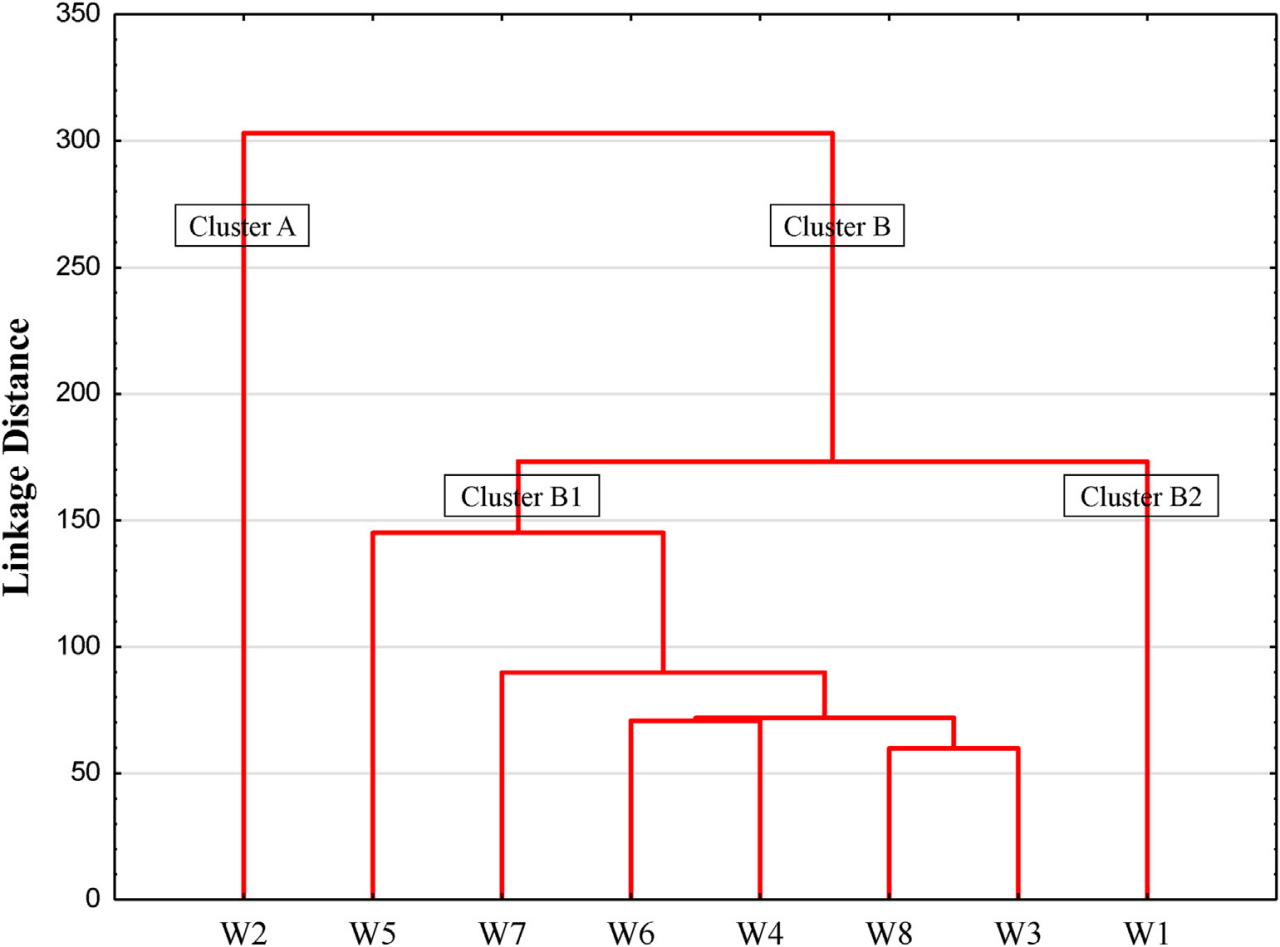
Cluster analysis.

The results of the cluster analysis represented in the form of a dendrogram show the similarity between the sampling points (Figure 12). The CA has been reported to be effective for classifying true groups of data based on their similarities (Chen et al., 2018). The dendrogram was composed of two main groups or clusters (Figure 12). Cluster A was composed by the sampling point W2. It is the first sampling point in the gold mining site. The values of water parameters were not very high in this point. Clusters B1 and B2 made up the second cluster (Cluster B). The sampling point W5 and all other sampling points in the gold mining site and downstream made up cluster B1. These sampling points had the highest values of water parameters, particularly sampling point W6 with high metal concentrations. At this point, the metal concentrations exceeded the limits set by the World Health Organization, 2011, World Health Organization, 2017. These metals were found in water samples as a results of gold mining activities and the weathering of specific minerals in gold veins. The cluster B2 was made up of sampling point W1, located upstream of the gold mining site and had the lowest measured values on a regular basis. The cluster analysis depicts the spatial impact of gold mining on the quality of the Lom River.

### 3.6 Geostatistical modeling

**Figure 13:**
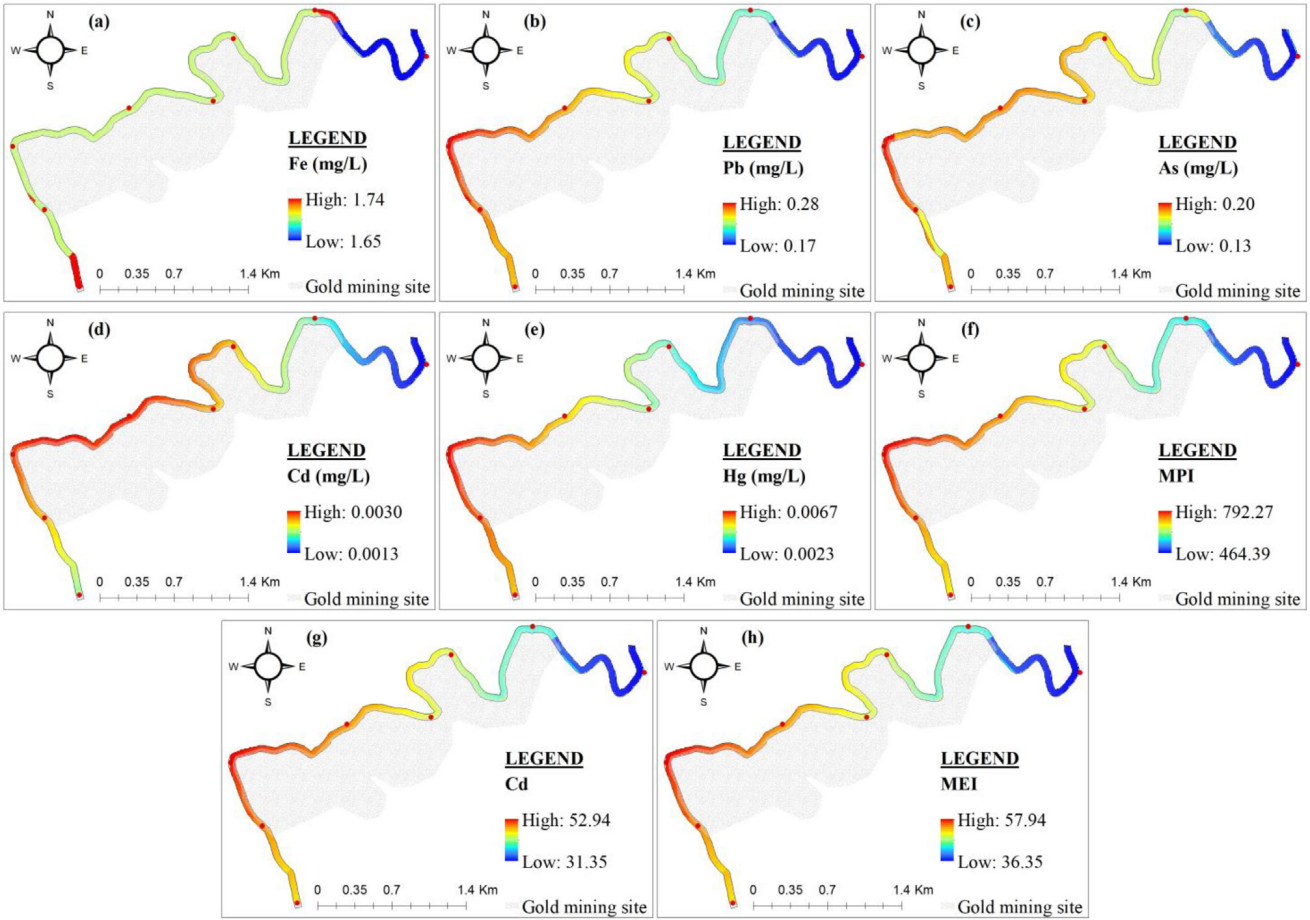
Kriged maps of metals and pollution indices: (a) Fe; (b) Pb; (c) As; (d) Cd; (e) Hg; (f) metal pollution index (MPI); (g) contamination degree (Cd) and (h) metal evaluation index (MEI).

Using ordinary kriging interpolation, predictive maps of metals and pollution indices were generated (Figure 13). The use of geostatistic indicator allows mapping of the prediction. The maps obtained with kriging could be useful for decision makers as they are easy to interpret. The prediction maps give concentrations on un-sampled points. All the studied metals and pollution indices had lower values upstream of the gold mining site (Figure 13). The values of Fe (1.65–1.74 mg/L), Pb (0.15–0.25 mg/L), and As (0.14–0.19 mg/L) throughout the Lom River in the mining site and downstream were uniform (Figures 13a, 13b and 13c). The concentrations of Cd (0.001–0.003 mg/L), Hg (0.0023–0.0067 mg/L), MPI (464.39–792.27), Cd (31.35–52.94) and MEI (36.35–57.94) increased in the gold mining site and decreased downstream of the mining site (Figures 13d, 13e, 13f, 13g and 13h). The increase in Hg concentrations in the studied section of the Lom River could be attributed to the use of mercury for gold washing during the exploitation.

## 4 Conclusions

The impact of gold mining on the quality of the Lom River at Gankombol (Adamawa Cameroon) was assessed in this paper. The study was conducted during the dry and the rainy seasons. The Gankombol sector was made up of metamorphic rocks: gneisses and micaschists; intrusive rocks: granodiorites and granites and quartz and quartzo-feldspathic veins, tourmaline quartz veins, and tourmaline and wolframite quartz veins. The waters of the Lom River draining the gold mining site at Gankombol were acidic to near-neutral (5.3–6.9) very turbid (117–510 NTU) with high level of total suspended solids (22.89–471 mg/L). The Lom River was unfit for human consumption (WQI >300) and polluted in Fe, Pb and As with mean concentrations (1.7, 0.2 and 0.2 mg/L) higher than those fixed by the WHO standards (0.3, 0.01 and 0.01 mg/L). The multivariate statistical analysis suggested strong linear associations between EC-TDS (R = 0.77), EC-Na^+^ (R = 0.78), TDS-Na^+^ (R = 0.76), Pb–As (R = 0.84), Cl^−^ –SO_4_^2-^ (R = 0.80) and TSS-Cd (R = 0.80) indicating that they had common sources. The geostatistical modeling revealed that metals and pollution indices had lower values upstream of the gold mining site. The subject was relevant in the field of research because this study revealed a major environmental problem which is linked to the pollution of the rivers draining the gold mining operations. It is therefore a subject of particular concern given the potential dangers to human health and aquatic life. In addition, this study is useful for political and administrative decision-makers, as it shows environmental impacts of gold mining in the quality of the Lom River at Gankombol. Therefore, they must take urgent action to implement adequate and sustainable remedial measures in order to reduce the negative impacts of mining activities in auriferous areas. For future works, it would be interesting to (i) carry out multi-year monitoring of the impacts of gold mining on the water quality of the Lom River; (ii) determine the concentrations of other metals such as Cu, Zn, Se and Mn in the Lom River and (iii) assess the impact of gold mining on groundwater, sediments, fauna, flora and air and local populations.

## Declarations

### Author contribution statement

Mouhamed Ngounouno Ayiwouo: Conceived and designed the experiments; Performed the experiments; Analyzed and interpreted the data; Contributed reagents, materials, analysis tools or data; Wrote the paper.

Fadimatou Ngounouno Yamgouot: Conceived and designed the experiments; Performed the experiments; Analyzed and interpreted the data; Contributed reagents, materials, analysis tools or data; Wrote the paper.

Luc Leroy Ngueyep Mambou: Analyzed and interpreted the data; Contributed reagents, materials, analysis tools or data; Wrote the paper.

Sifeu Takougang Kingni: Analyzed and interpreted the data; Contributed reagents, materials, analysis tools or data; Wrote the paper.

Ismaila Ngounouno: Analyzed and interpreted the data; Contributed reagents, materials, analysis tools or data; Wrote the paper.

### Funding statement

This research did not receive any specific grant from funding agencies in the public, commercial, or not-for-profit sectors.

### Data availability statement

Data will be made available on request.

### Declaration of interest’s statement

The authors declare no conflict of interest.

### Additional information

No additional information is available for this paper.
